# Exome array analysis of rare and low frequency variants in amyotrophic lateral sclerosis

**DOI:** 10.1038/s41598-019-42091-3

**Published:** 2019-04-11

**Authors:** Annelot M. Dekker, Frank P. Diekstra, Sara L. Pulit, Gijs H. P. Tazelaar, Rick A. van der Spek, Wouter van Rheenen, Kristel R. van Eijk, Andrea Calvo, Maura Brunetti, Philip Van Damme, Wim Robberecht, Orla Hardiman, Russell McLaughlin, Adriano Chiò, Michael Sendtner, Albert C. Ludolph, Jochen H. Weishaupt, Jesus S. Mora Pardina, Leonard H. van den Berg, Jan H. Veldink

**Affiliations:** 1 Department of Neurology, Brain Center Rudolf Magnus, University Medical Center Utrecht, Utrecht, The Netherlands; 2 Rita Levi Montalcini’ Department of Neuroscience, ALS Centre, University of Torino, Turin, Italy; 3 KU Leuven - University of Leuven, Department of Neurosciences, Experimental Neurology and Leuven, Research Institute for Neuroscience and Disease (LIND), B-3000 Leuven, Belgium; 4 VIB, Vesalius Research Center, Laboratory of Neurobiology, Leuven, Belgium; 5 University Hospitals Leuven, Department of Neurology, Leuven, Belgium; 6 Academic Unit of Neurology, Trinity College Dublin, Trinity Biomedical Sciences Institute, Dublin, Ireland; 7 Department of Neurology, Beaumont Hospital, Dublin, Ireland; 8 Population Genetics Laboratory, Smurfit Institute of Genetics, Trinity College Dublin, Dublin, Ireland; 9 Institute of Clinical Neurobiology, University of Würzburg, Würzburg, Germany; 10 Department of Neurology, Ulm University, Ulm, Germany; 11 ALS Unit, Hospital San Rafael, Madrid, Spain

## Abstract

Amyotrophic lateral sclerosis (ALS) is a fatal neurodegenerative disease that affects 1 in ~350 individuals. Genetic association studies have established ALS as a multifactorial disease with heritability estimated at ~61%, and recent studies show a prominent role for rare variation in its genetic architecture. To identify rare variants associated with disease onset we performed exome array genotyping in 4,244 cases and 3,106 controls from European cohorts. In this largest exome-wide study of rare variants in ALS to date, we performed single-variant association testing, gene-based burden, and exome-wide individual set-unique burden (ISUB) testing to identify single or aggregated rare variation that modifies disease risk. In single-variant testing no variants reached exome-wide significance, likely due to limited statistical power. Gene-based burden testing of rare non-synonymous and loss-of-function variants showed *NEK1* as the top associated gene. ISUB analysis did not show an increased exome-wide burden of deleterious variants in patients, possibly suggesting a more region-specific role for rare variation. Complete summary statistics are released publicly. This study did not implicate new risk loci, emphasizing the immediate need for future large-scale collaborations in ALS that will expand available sample sizes, increase genome coverage, and improve our ability to detect rare variants associated to ALS.

## Introduction

Amyotrophic lateral sclerosis (ALS) is a rapidly progressing and fatal neurodegenerative disease with an estimated lifetime risk of approximately 1 in 350^1^. The central hallmark of the disease is dysfunction and gradual loss of upper and lower motor neurons, resulting in progressive muscle weakness and eventual death due to respiratory failure. Median survival is three to five years after symptom onset^2,3^. A subset of patients shows clinical signs of extramotor system involvement, most notably cognitive impairment^4^. There is no cure to date, and the sole effective drug, riluzole, only extends survival by approximately three months^5^.

ALS is considered a complex disease to which both environmental and genetic risk factors contribute to disease susceptibility. Twin-based heritability is estimated at approximately 61%^6^. Over the past decades multiple genes and genomic regions have been implicated in ALS via linkage studies, genome-wide association studies (GWAS), and more recently, large scale sequencing efforts^7^. A recent GWAS in ALS, in approximately 14,000 cases and 30,000 controls, identified a total of 7 loci associated to the disease. The top loci together explain 0.2% of the estimated 8.5% SNP-based heritability; heritability estimates across all SNPs indicate that the bulk of the SNP-based heritability is captured in low-frequency (defined as minor allele frequency (MAF) 1–10%) variants beneath genome-wide significance^8^. These findings are consistent with a disease architecture in which rare and low-frequency variants play an important role^9^.

Imputation of low-frequency (MAF 0.5–5.0%) variants in genotyping data has become more accurate with the increasing public availability of large reference panels, and with the combination of public reference panels with study-specific reference panels^10,11^. High quality imputation of rare variants, (MAF < 0.5%), however, has remained challenging^12,13^. The exome array provides a low-cost alternative to large-scale exome or genome sequencing, albeit with lower resolution. In this largest exome-wide study in ALS to date, we used the exome array to investigate the role of low-frequency and rare variants by genotyping over 240,000 primarily functional coding variants in 7350 ALS patients and controls drawn from different European cohorts. Using this approach, we aimed to further elucidate the role of these low-frequency and rare variants in the genetic landscape of ALS.

## Results

Analyses comprised 4,244 cases and 3,106 controls from 6 European countries. Of 242,901 genotyped sites, 233,331 sites passed quality control, of which 100,896 were non-monomorphic in cases and controls. A breakdown of the included samples per country is provided in Supplementary Table 1.

### Single-variant testing

**Figure 1:**
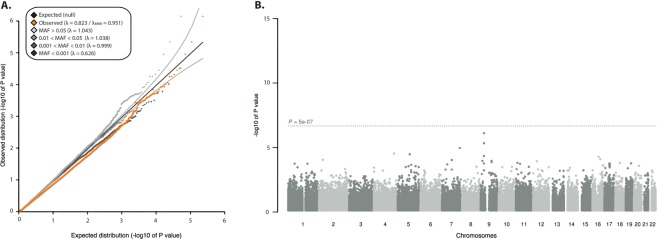
Quantile-quantile plot and Manhattan plot of p-values of the single-variant association analysis. (**A**) Quantile-quantile plot of single-variant association analysis using logistic regression binned by minor allele frequency. (**B**) Manhattan plot of p-values of exome-wide association testing comprising 7350 individuals (4,244 cases and 3,106 controls) and 100,896 non-monomorphic variants. The *x* axis depicts chromosomal position and the *y* axis shows the significance of association derived by logistic regression. The dotted line corresponds to the exome-wide significance threshold of p = 5 × 10^−7^).

We performed single-variant testing using logistic regression, adjusting for sex and population principal components, and assuming an additive model. We found the genomic inflation factor (λ_GC_) to be 0.823 (Fig. 1A), reflecting the high prevalence of rare (i.e., MAF < 0.5%) variants in the data and therefore low power to detect effects at these rare alleles. No variants reached exome-wide significance (p < 5 × 10^−7^; Fig. 1B). Multiple top-associated signals (MAF 0.23–0.29) were located on chromosome 9 (lowest p-value 1.72 × 10^−6^; Supplementary Table 2). These signals represent the large intronic hexanucleotide repeat expansion in the gene *C9orf72*, the most common genetic cause of ALS identified to date^14,15^. The strongest associated rare variants (MAF < 0.5%), rs200161705 (odds ratio (OR) = 2.74, p = 5.76 × 10^−5^) and rs181906086(OR = 0.35, p = 1.68 × 10^−4^), are both missense variants as annotated by Variant Effect Predictor^16^ and are located in the genes *NEK1* and *CAPN14* respectively (Supplementary Table 2).

Previous work has shown that, in testing rare variation down to minor allele count <400, the Firth test has the best combination of type I error and power in balanced and moderately unbalanced case-control studies^17^. We therefore repeated our analysis using Firth logistic regression and found the results to be highly consistent with our initial regression results (data not shown).

Supplementary Table 3 shows the results from one of the largest ALS GWAS to date (Van Rheenen *et al*.^8^) compared to the results from our single variant analysis^8^.

### Gene-based analysis

**Figure 2:**
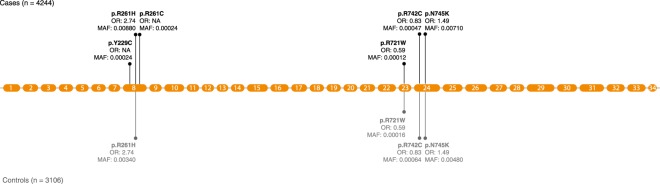
Gene mutation plot of *NEK1*. The orange circles represent exons relative to their size in basepairs. The vertical lines represent the distribution of SNVs across *NEK1* in the gene-based test (SKAT-O), with their corresponding amino acid change, minor allele frequency (MAF) and odds ratio (OR) in the single-variant analysis.

Next, to test for an association between aggregated rare variants clustering in genes and ALS, we performed gene-based analysis using the unified optimal sequence kernel association test (SKAT-O)^18^. As SKAT-O is a combination of burden and variance statistics, the test retains discovery power under different genetic architecture models (i.e., by varying the proportion of causal variants within the gene that either have the same direction of effect or presence of both protective and deleterious variants, and then testing for association). We observed no evidence for residual population stratification based on the standardized genomic inflation factor λ_GC_ of 1.016 after correction for principal components, sex and cohort (Supplementary Figure 1). No individual gene surpassed the predefined Bonferroni-corrected significance threshold (p = 3.45 × 10^−6^, after adjusting for 14,488 genes). For the top genes most associated to ALS (p < 1 × 10^−3^; Table 1), we calculated exact p-values using 500,000 case-control permutations. The strongest signal in the gene-based burden test using SKAT-O was *NEK1* (p = 1.21 × 10^−5^; Fig. 2). For the majority of the top associating genes repeating the analysis using Firth test showed highly concordant results (Table 1). Conditioning the gene-based analysis on variants in that gene present as top hit in the single-variant analysis resulted in an expected rise in p-value, due to loss in power after conditioning on the variant with the highest MAF (Supplementary Table 4).

**Table 1:** Top genes associated to ALS, as found through gene-based burden testing.

Gene	Chr	Start (bp)	End (bp)	Nominal p value SKAT-O	Exact p value SKAT-O	Nominal p value Firth test	Number of variants
*NEK1*	4	170314421	170533778	2.73 × 10^−5^	1.21 × 10^−5^	3.06 × 10^−5^	6
*ZNF287*	17	16453626	16472520	3.08 × 10^−5^	3.21 × 10^−5^	2.06 × 10^−5^	2
*CAPN14*	2	31395922	31456724	2.16 × 10^−4^	1.73 × 10^−4^	1.18 × 10^−3^	5
*IRF8*	16	85932774	85956212	2.50 × 10^−4^	2.25 × 10^−4^	1.34 × 10^−3^	4
*F10*	13	113777113	113803843	5.94 × 10^−4^	4.77 × 10^−4^	5.20 × 10^−4^	4
*PTPRB*	12	70910630	71031220	7.10 × 10^−4^	5.74 × 10^−3^	0.37	21
*SYNM*	15	99645286	99675800	7.55 × 10^−4^	6.14 × 10^−3^	5.05 × 10^−4^	8
*SGK3*	8	67624653	67774257	7.90 × 10^−4^	7.00 × 10^−3^	8.52 × 10^−4^	2

Results of gene-based burden testing using SKAT-O and Firth test, results limited to genes exceeding p < 1 × 10^−3^ using SKAT-O. Positions given for human build 37. Exact p values generated from 500,000 case-control permutations.

Resolution for gene-based analysis was assessed by comparing the number of missense and loss-of-function variants in previously identified ALS genes (i.e., *SOD1*, *FUS*, *TARDBP*, *KIF5A*, *NEK1* and *C21orf2*) present in ExAC to the number of variants tested in our gene-based analysis (Supplementary Table 5). SKAT-O analysis in these genes comprised a maximum of 6 variants (*NEK1*; p = 1.21 × 10^−5^).

### Individual set-unique burden analysis

Previous studies have indicated that in some complex diseases, disease-affected individuals carry an excess number of missense and nonsense variants^19^. Therefore, we next sought to investigate the exome-wide burden per individual of particular classes of variants by estimating the individual set-unique burden (ISUB) for each individual. We first annotated all variants available for analysis. Of the 24,844 variants carried only by the 4,244 ALS cases, 23,231 variants are nonsynonymous and 6,600 are deleterious, according to the CONDEL algorithm^20^; these variants comprise the ‘set-unique’ variants in cases. Of the 11,856 set-unique variants in the 3,106 controls, 11,088 are nonsynonymous and 3,135 are deleterious. We used CONDEL to assign deleteriousness scores to each variant and then summed and normalized these scores. Finally, for all set-unique nonsynonymous and loss-of-function variants (called the ‘NS’ group of variants) as well as for the observed loss-of-function variants predicted to be deleterious (the ‘DEL’ group of variants), we calculated the exome-wide individual set-unique burden (ISUB) per individual.

We found the ISUB scores based on the NS group of variants to be significantly higher in ALS patients compared to controls (p = 9.6 × 10^−137^). We observed no such difference for ISUB calculated from the DEL group of variants (p = 0.21) (Table 2, Supplementary Figure 3). To control for the case-control imbalance in our sample (case-control ratio ~1.36:1) we repeated the ISUB analysis in balanced cohorts only (i.e., including 5,069 individuals from the Dutch, Belgian, and Irish cohorts with case-control ratios nearing 1:1). In this balanced subset, we observed no significant difference in ISUB score based either on nonsynonymous variants (p = 0.37) or on deleterious variants (p = 0.51, Table 2, Supplementary Figures 2 and 4).

**Table 2:** Results individual set unique burden analysis.

Variant type	Phenotype	All Cohorts				Balanced Cohorts			
		No. of individuals	No. of SNVs	ISUB score (mean/median/sd)	p value	No. of individuals	No. of SNVs	ISUB score (mean/median/sd)	p value
DEL	*Case*	4244	6600	1.58/1.23/1.57	0.21	2489	4201	1.47/1.20/1.62	0.51
	*Control*	3106	3135	1.38/1.13/1.49		2580	4025	1.42/1.21/1.20	
NS	*Case*	4244	23,231	4.33/3.49/4.16	9.6 × 10^−137^	2489	14,831	3.97/3.43/4.55	0.37
	*Control*	3106	11,088	2.30/1.89/2.10		2580	14,310	3.83/3.49/2.82	

Results given for analysis comprising all individuals (all cohorts; N = 7350) and for a subset of samples comprising balanced case-control cohorts only (balanced cohorts; samples from The Netherlands, Belgium and Ireland, N = 5069). P values given for logistic regression with first six principal components and country of sample origin included as covariates. DEL = deleterious variants, NS = all nonsynonymous and loss-of-function variants.

We additionally confirmed that the differing number of observed variants per individual (N = 7 and N = 4 respectively, p = 1.38^−276^) in the imbalanced (i.e., complete) dataset drove the significant difference in set-unique burden between cases and controls (Supplementary Table 6). Balancing the case-control ratio eliminated this difference (N = 7 for nonsynonymous variants in both the case and control datasets, p = 0.19) (Supplementary Table 6, Supplementary Figure 5). The scores of deleteriousness per variant, as measured by CONDEL, did not differ between cases and controls for the imbalanced dataset (p = 0.58) and the balanced dataset (p = 0.87) (Supplementary Table 7, Supplementary Figure 5). Repeating the analysis after removing outlying scores (defined as >5 standard deviations from set mean ISUB score) or logarithmic transformation of the data did not change the results.

## Discussion

Using the exome array in 7,350 ALS cases and matched controls, we sought to investigate the role of low-frequency and rare variants in the etiology of ALS. Despite being the largest study of rare variants in ALS to date, no associations reached the predefined significance levels in single-variant association testing, gene-based burden testing and exome-wide individual set-unique burden (ISUB) testing. The strongest associating signals have previously been linked to ALS.

In accordance with other exome array studies, we had minimal power to find significant associations at rare, modest effect variants. At a minor allele frequency (MAF) of 1%, variants with large effect sizes (odds ratio >2.3) could be detected with 80% power; larger effect sizes are necessary to have sufficient power (>80%) for variants with lower frequency. Because the exome array content is nearly entirely comprised of rare variants that, by definition, typically have low LD with other variants, our power to detect other associated variants with ALS through LD-tagging was also low. Given our results, we can conclude that none of the rare variants captured on the exome array are large-effect variants associated with ALS.

For gene-based burden testing our analysis yielded sufficient power to detect genes with a large proportion of causal variants (≥50%) contributing to disease risk (including genes with ≤25% protective variants). For genetic architectures with lower percentages of causal variants and higher percentages of protective variants, power was low. Different genes associated to ALS likely contain different genetic architectures. For example, previous work showed that mutations in several ALS-related genes predominantly affect specific regions of the gene (e.g. mutations causing exon 7 skipping in *FUS* and mutations primarily located at the C-terminal cargo-binding region in *KIF5A)*, whereas in other genes (e.g. *SOD1*) mutations are more dispersed^21–24^. This architectural complexity makes selecting appropriate settings for power analyses difficult; while we can evaluate power for simpler architectures (e.g., genes containing mostly causal risk variation), it is difficult to evaluate power for a gene with a much more complex architecture (e.g., with both risk and protective variants that must occur on specific haplotypes and/or in combination with specific genetic backgrounds)^25^.

Additional limitations include the fact that of the ~240,000 variants present on the array; the majority were monomorphic in our dataset. Illustrative of this low resolution is the observation that for gene-based burden testing of *KIF5A*, a gene recently implicated in ALS pathogenesis, our dataset only included three variants. Two of these three variants are located outside the previously identified ALS-associated domain in the C-terminus of the protein^23,24^. As a result, we did not find an association between *KIF5A* and ALS. Our analyses also only include European-ancestry samples; given the strong geographic localization of rare variants, such a design leaves the many rare variants found only in non-European samples untested. Further, since most of the rare variants that have been identified in rare variant association studies to date have modest-to-weak effect sizes, much larger sample sizes or denser coverage of the genome are necessary in order to robustly implicate novel associations that increase risk of disease.

Despite power limitations, we can still draw several key conclusions. First, we confirm several of the strongest associations previously found in ALS, demonstrating the validity of our approach. The common variants associating most strongly with ALS in this study map to the gene *C9orf72*, a well-established genetic risk factor in ALS. These signals are driven by a massive hexanucleotide repeat (GGGGCC) expansion between non-coding exons 1a and 1b, an expansion which is the most frequent genetic cause of ALS discovered to date^26^. The strongest associated rare variant (rs200161705) located in *NEK1* was recently discovered as a risk variant in an inbred population in The Netherlands and subsequently replicated in an international cohort including ALS patients with and without a positive family history^27^. Since these initial findings, several studies have confirmed *NEK1* as an ALS risk gene^27–29^. In our study, *NEK1* was the top hit in the gene-based analysis, demonstrating how, in some instances, application of the exome array can be a more efficient approach than sequencing for finding large effect variants. Future studies that use arrays with both exome and common variant content, to allow for not only interrogation of rare coding variants but also interrogation of common variation through genotyping and imputation, will be well positioned to identify signals like that residing in *NEK1*.

Because *CAPN14* gene was among the top findings in both single-variant and gene-based analysis, it represents a tantalizing finding but one that requires additional follow-up in larger samples. *CAPN14* is a member of the calpain family; calpains are cysteine proteases that are activated by calcium, possibly through increased glutamatergic neuronal transmission. Calpains have been identified as regulators of axonal survival in injury-induced and developmental degeneration via necrotic and apoptotic pathways^30–32^ and an ALS mouse model has shown calpain inhibition to be neuroprotective^33^. Calpain14 is primarily expressed in esophageal mucosa and has also been associated with eosinophilic esophagitis^34,35^. Although an association between the calpain family and ALS is possible, future studies will be necessary to further establish and later unravel a potential role for the gene in disease etiology. While our ISUB analysis did not reveal an exome-wide increase in individual set-unique burden between cases compared to controls, our results do demonstrate the importance of careful study design, in particular of case-control studies seeking to investigate rare variation. A case-control imbalance, allowing for the detection of more rare variants in one group than the other, can induce spurious associations if not handled properly. An identical analysis of genome-wide individual burden of nonsynonymous and loss-of-function variants performed in schizophrenia found a difference between cases and controls in a comparatively smaller sample (N = 2,003)^19^. Despite the described genetic overlap of ALS and schizophrenia, the genetic architecture of schizophrenia is likely quite different from ALS^36^. GWAS in schizophrenia have revealed a prevalent role for common variants in the disease, and schizophrenia is also more heritable (h^2^~80%)^37^. Given the highly polygenic architecture of schizophrenia, an ISUB analysis in that phenotype may better capture the many disease-associated loci scattered across the genome. In contrast, the architecture of ALS may be less polygenic and/or more region-specific, and inclusion of exome-wide variation may contribute additional noise to the analysis, thus obscuring potential statistical signal. Additional studies that help to first pinpoint these potential localized signals are necessary to test this hypothesis.

With the lowering costs of high-coverage sequencing and the increasing availability of large reference panels that allow for imputation of tens of millions of low-frequency variants, high-quality studies of rare variants in ALS are expected in the coming years^38^. These future larger and more resolute studies are expected to allow additional data filtering in order to separate noise from true signal and will be better powered to accommodate the diverse genic architectures observed in ALS to date. Given that our analysis of functional coding variants in thousands of European ALS patients and controls did not implicate new risk loci, our analyses reaffirm the need for future studies of rare variants in ALS that are internationally collaborative, allowing for the collection of larger and more ancestrally diverse sample sizes and denser coverage of the genome. In order to facilitate large scale rare variant analyses summary statistics will be made publicly available through the Project MinE databrowser website at http://databrowser.projectmine.com/.

## Methods

### Study population

All 4495 patients and 3227 controls included in this study were recruited at specialized neuromuscular centers in Belgium, Germany, Ireland, Italy, Spain and the Netherlands. Patients were diagnosed with possible, probable or definite ALS according to the 1994 El-Escorial criteria^39^. All controls were free of neuromuscular diseases and matched for age, sex and geographical location.

### Genotyping and quality control

We conducted genotyping using Illumina HumanExome-12v1 BeadChips in accordance with the manufacturer’s protocol. A description of the exome chip design is available from http://genome.sph.umich.edu/wiki/Exome_Chip_Design. We applied the GenTrain 2.0 clustering algorithm for genotype calling as implemented in the Illumina GenomeStudio software package. Initial genotype calls were made based on the HumanExome cluster file provided by Illumina. More accurate cluster boundaries were determined based on the actual study data by exclusion of samples with a GenCall quality score in the lowest 10th percentile of genotyped variants (p10GC < 0.38) or call rate <0.99. The final genotype calls of the whole dataset were obtained using these more precise cluster boundaries. We then performed additional sample and variant quality control (QC) was performed using PLINK 1.9, excluding all samples with missing call rates higher than 5% or sex discordance^40,41^. A subset of independent, high quality SNPs (non-AT/CG, autosomal variants not located in high linkage disequilibrium (LD) regions with minor allele frequency (MAF) > 0.05, genotyping rates >99%, R^2^ < 0.05 and Hardy-Weinberg equilibrium p values > 1 × 10^−3^) was used to determine heterozygosity rates, population stratification, intersample relatedness and sample duplication. Closely related and duplicated samples (pi-hat > 0.2) and samples failing heterozygosity checks (>4 standard deviations) were removed. To assess population stratification, we calculated principal components on all remaining individuals and HapMap3 individuals using EIGENSTRAT^42,43^. We removed population outliers (defined as >10 standard deviations from the HapMap CEU mean on PC 1–4) (Supplementary Figure 8a). Repeating principal component analysis with data from The 1000 Genome Project did not result in the removal of additional population outliers (Supplementary Figure 8b). Non-autosomal variants and variants with haploid heterozygous calls, call rate <98%, deviation from Hardy-Weinberg equilibrium in controls (p < 1 × 10^−6^) and biased missingness between cases and controls (p < 5 × 10^−3^) were removed from the dataset. The final dataset comprised a total of 7,350 individuals (4,244 cases and 3,106 controls) and 233,331 variants.

### Statistical analyses

We performed single-variant association testing in PLINK 1.9 using logistic regression under an additive genetic model^40,41^. Covariates were selected based on significant correlation to the phenotype using logistic regression. To correct optimally for population stratification and batch effects due to sample handling, we used the first five principal components, sex and country of sample origin as covariates. We tested all non-monomorphic variants in both cases and controls (N = 100,896). We corrected for multiple testing using a Bonferroni adjusted p value threshold (p = 0.05/100,896 = 4.95 × 10^−7^), which nears the exome-wide significance threshold of 5 × 10^−7^. Genomic inflation factors were calculated using R v3.2.2 (http://www.r-project.org). Since previous work showed that Firth test has the best combination of type I error and power in balanced and moderately unbalanced studies of rare variant (i.e. variants with a minor allele count <400) we repeated the single-variant analysis using Firth logistic regression in R v3.4.1. as implemented in the logistf package.

Gene-based analysis was conducted using the unified optimal sequence kernel association test (SKAT-O) as implemented in the R-package *SKAT*^44^. Variants were functionally annotated using Ensembl Variant Effect Predictor (VEP)^16^. All non-synonymous and loss-of-function variants with a MAF < 0.01 and minor allele count >1 were included. Genes containing one annotated variant were excluded. The model was run with sex, the first five principal components and country included in the model as covariates. Exact p-values were generated from 500,000 case/control permutations for hits with a nominal p-value < 0.001. Results were considered significant after Bonferroni correction for number of tests (p = 0.05/14,488 = 3.45 × 10^−6^). Burden testing was repeated using Firth logistic regression in R v3.4.1.

To investigate the exome wide burden of rare single nucleotide variants (SNVs) in ALS cases compared to controls an individual set-unique burden (ISUB) analysis was performed as described previously^19^. Only variants uniquely present in controls or uniquely present in cases were selected for this analysis. Allele frequencies were compared to the non-Finnish European cohort in ExAC and variants with a MAF higher than 0.5% were removed^45^. Deleteriousness of the variants was assessed using the CONDEL algorithm, which assigns a weighted average of normalized scores from multiple prediction tools to each coding variant, as well as a ‘neutral’ or ‘deleterious’ label^20^. Non-scored loss-of-function variants were assigned a deleterious label and the maximum score occurring in the corresponding dataset. The final individual burden score was computed by summing the scores for all observed non-synonymous and loss-of-function set-unique variants (NS) as well as for the set-unique variants predicted to be deleterious (DEL).

We assigned scores under a dominant model, assigning a score only once in case of a homozygous genotype. The analysis was first performed in the complete dataset comprising 4,244 cases and 3,106 controls. Since the case-control ratio is an important determinant in whether a variant appears to be unique, we repeated all analyses in a balanced case-control subset of Dutch, Belgian and Irish samples (2,489 cases and 2,580 controls). Differences in ISUB scores between cases and controls were assessed using logistic regression with the first five principal components and country included in the model as covariates. To reduce the effect of outlying scores on the results the analyses were repeated with outlying scores (>5 standard deviations from set mean) removed as well as with logarithmic transformation of the data. R version 3.2.2 was used for all statistical analyses (http://www.r-project.org).

### Power analysis

To estimate detectable effect sizes given the low frequencies of rare single nucleotide variants, (Supplementary Fig. 6), we performed power analysis for single-variant associating testing using the online Genetic Power Calculator (http://zzz.bwh.harvard.edu/gpc/)^46^.

Power analysis for gene-based burden analysis using SKAT-O as implemented in the R-package *SKAT* revealed sufficient power (>86% at 200 simulations) to detect genes with a large proportion (≥50%) of causal (i.e., risk-increasing) variants among rare variants (defined as MAF < 0.01) contributing to disease risk, including genes with ≤25% protective (i.e., risk-decreasing) variants (Supplementary Figure 7) (see package documentation for information regarding assumptions, default settings and methods at https://CRAN.R-project.org/package=SKAT).

### Ethics statement

The study was approved by the Medical Ethical Committee from the University Medical Center Utrecht, The Netherlands. Also, the present study followed study protocols approved by Medical Ethical Committees for each of the participating institutions. Written informed consent was obtained from all participating individuals. All methods were performed in accordance with the relevant national and international guidelines and regulations.

## Supplementary information

Supplementary information

## Data Availability

Summary statistics for this study are publicly available through the Project MinE Data Browser at http://databrowser.projectmine.com/.
