# Supplementary material for: Exome array analysis of rare and low frequency variants in amyotrophic lateral sclerosis
Source: Sci Rep. 2019 Apr 11;9:5931. doi: 10.1038/s41598-019-42091-3 (PMC6459905; doi:10.1038/s41598-019-42091-3)
Supplement: Supplementary file 1 — Supplementary information [file 41598_2019_42091_MOESM1_ESM.pdf]

Exome array analysis of rare and low frequency variants in amyotrophic lateral sclerosis.

Annelot M. Dekker[1], Frank P. Diekstra[1], Sara L. Pulit[1], Gijs H.P. Tazelaar[1], Rick A. van der Spek[1], Wouter van Rheenen[1], Kristel R. van Eijk[1], Andrea Calvo[2], Maura Brunetti[2], Philip Van Damme[3,4,5], Wim Robberecht[3,4,5], Orla Hardiman[6,7], Russell McLaughlin[8], Adriano Chiò[2], Michael Sendtner[9], Albert C. Ludolph[10], Jochen H. Weishaupt[10], Jesus S. Mora Pardina[11], Leonard H. van den Berg[1#], Jan H Veldink[1#*]

1. Department of Neurology, Brain Center Rudolf Magnus, University Medical Center Utrecht, Utrecht, The Netherlands.

2. 'Rita Levi Montalcini' Department of Neuroscience, ALS Centre, University of Torino, Turin, Italy.

3. KU Leuven - University of Leuven, Department of Neurosciences, Experimental Neurology and Leuven. Research Institute for Neuroscience and Disease (LIND), B-3000 Leuven, Belgium.

4. VIB, Vesalius Research Center, Laboratory of Neurobiology, Leuven, Belgium.

5. University Hospitals Leuven, Department of Neurology, Leuven, Belgium.

6. Academic Unit of Neurology, Trinity College Dublin, Trinity Biomedical Sciences Institute, Dublin, Republic of Ireland.

7. Department of Neurology, Beaumont Hospital, Dublin, Republic of Ireland.

8. Population Genetics Laboratory, Smurfit Institute of Genetics, Trinity College Dublin, Dublin, Republic of Ireland.

9. Institute of Clinical Neurobiology, University of Würzburg, Würzburg, Germany.

10. Department of Neurology, Ulm University, Ulm, Germany.

11. ALS Unit, Hospital San Rafael, Madrid, Spain

[#] Co-last authors.

Contents Supplementary Information

Supplementary Figure 1: Quantile-quantile plot of gene-based analysis.
Supplementary Figure 2: Set-unique SNVs.
Supplementary Figure 3: Individual set unique burden analysis in all cohorts.
Supplementary Figure 4: Individual set unique burden analysis in balanced cohorts.
Supplementary Figure 5: Breakdown individual set unique burden score.
Supplementary Figure 6: Power plot of single variant analysis.
Supplementary Figure 7: Power plot of gene-based burden test.
Supplementary Figure 8: PCA plots of population structure.
Supplementary Table 1: Study population.
Supplementary Table 2: Single variant association test using logistic regression.
Supplementary Table 3: Single nucleotide variant association test results for previously identified ALS variants.
Supplementary Table 4. Variant characteristics gene-based burden test *NEK1* and *CAPN14*.
Supplementary Table 5: Exome array resolution for previously identified ALS genes.
Supplementary Table 6: Comparison of set-unique variant count per individual.
Supplementary Table 7: Comparison of CONDEL score per variant.

Supplementary Figure 1. Quantile-quantile plot gene-based analysis.

QQ-plot of gene-based analysis using SKAT-O.

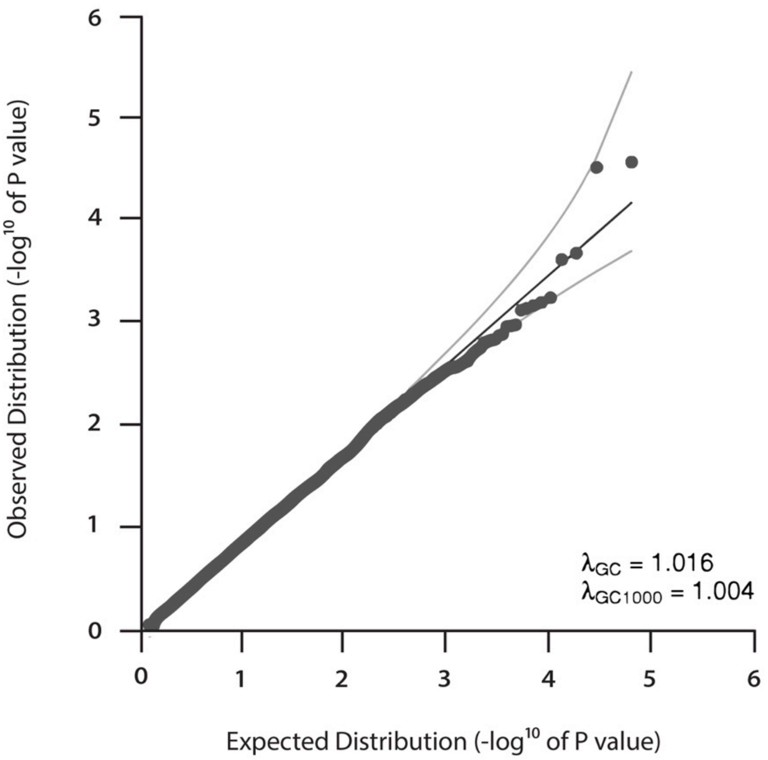

Supplementary Figure 2. Set-unique SNVs

Histogram depicting the set-unique variants selected for ISUB analysis in cases and controls from all cohorts combined (cases N = 4244, controls N = 3106) and from balanced cohorts only (cases N = 2489, controls N = 2580). DEL = deleterious variants, NS = all non-synonymous and loss-of-function variants.

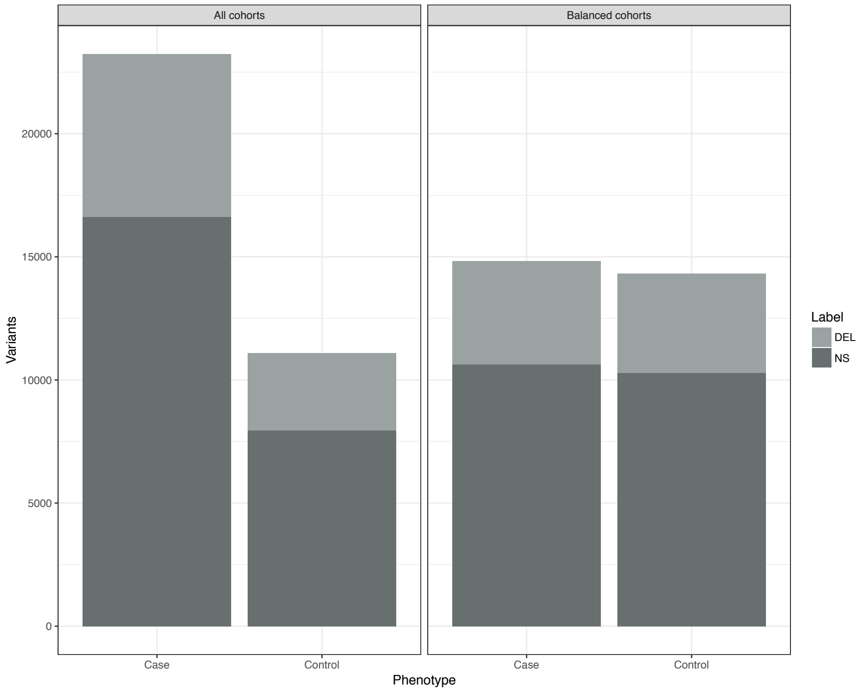

Supplementary Figure 3. Individual set-unique burden analysis in all cohorts.

Boxplots of ISUB scores in analysis comprising all individuals (N = 7350) and individuals per included cohort for (A) deleterious variants only and (B) all non-synonymous and loss-of-function variants.

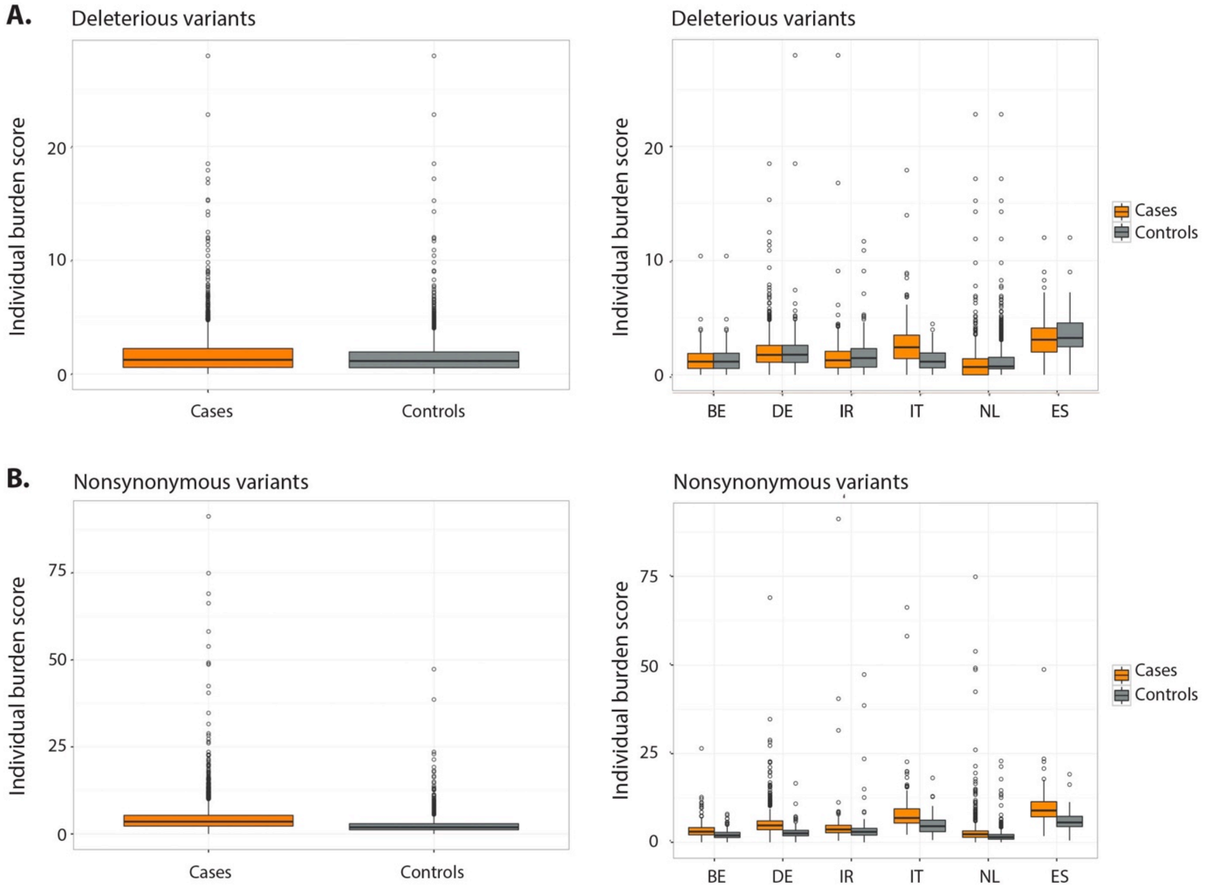

Supplementary Figure 4. Individual set unique burden analysis in balanced cohorts. Boxplots of ISUB scores in analysis comprising balanced case-control cohorts (samples from The Netherlands, Belgium and Ireland, N = 5069) and individuals per included cohort for (A) deleterious variants only and (B) all non-synonymous and loss-of-function variants.

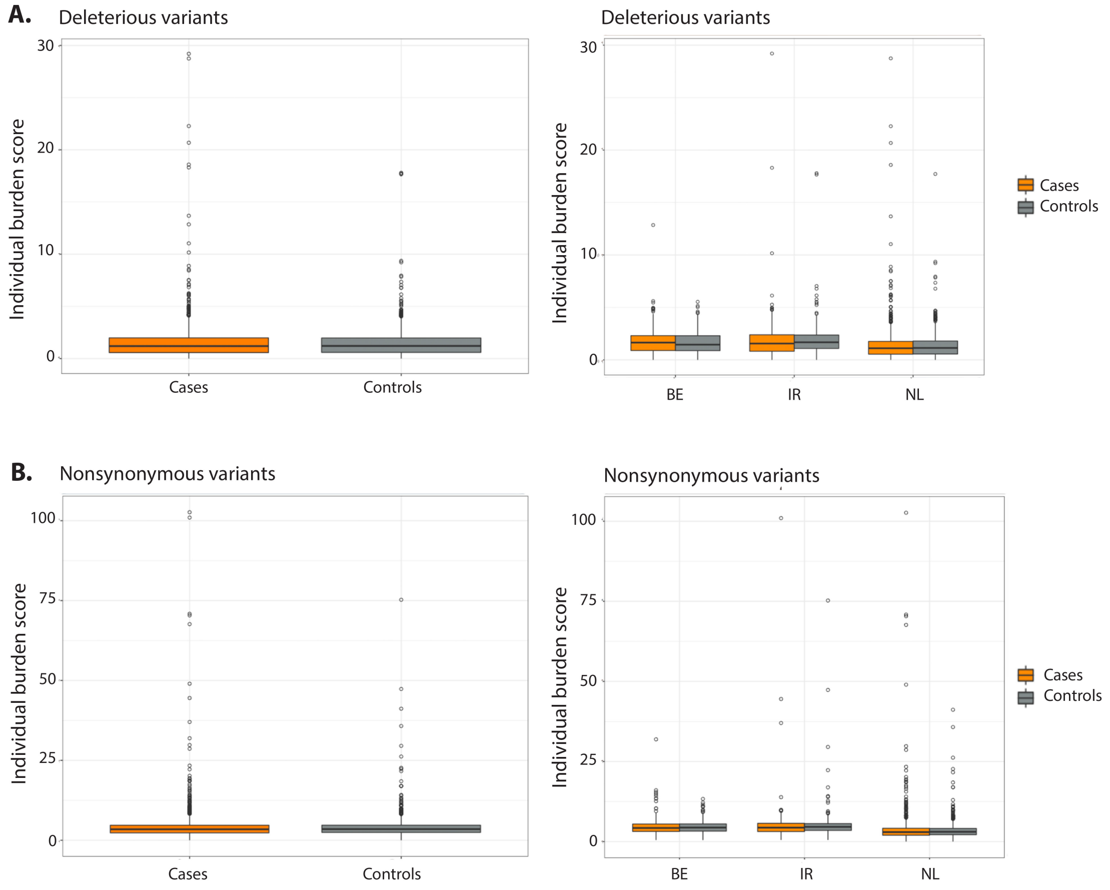

Supplementary Figure 5. Breakdown individual set unique burden score.

Analyses comprising all individuals (all cohorts; N = 7350) and subset of samples comprising balanced case-control cohorts only (balanced cohorts; samples from The Netherlands, Belgium and Ireland, N = 5069): (A) boxplot of CONDEL score per variant; (B) boxplot of set-unique variant count per individual.

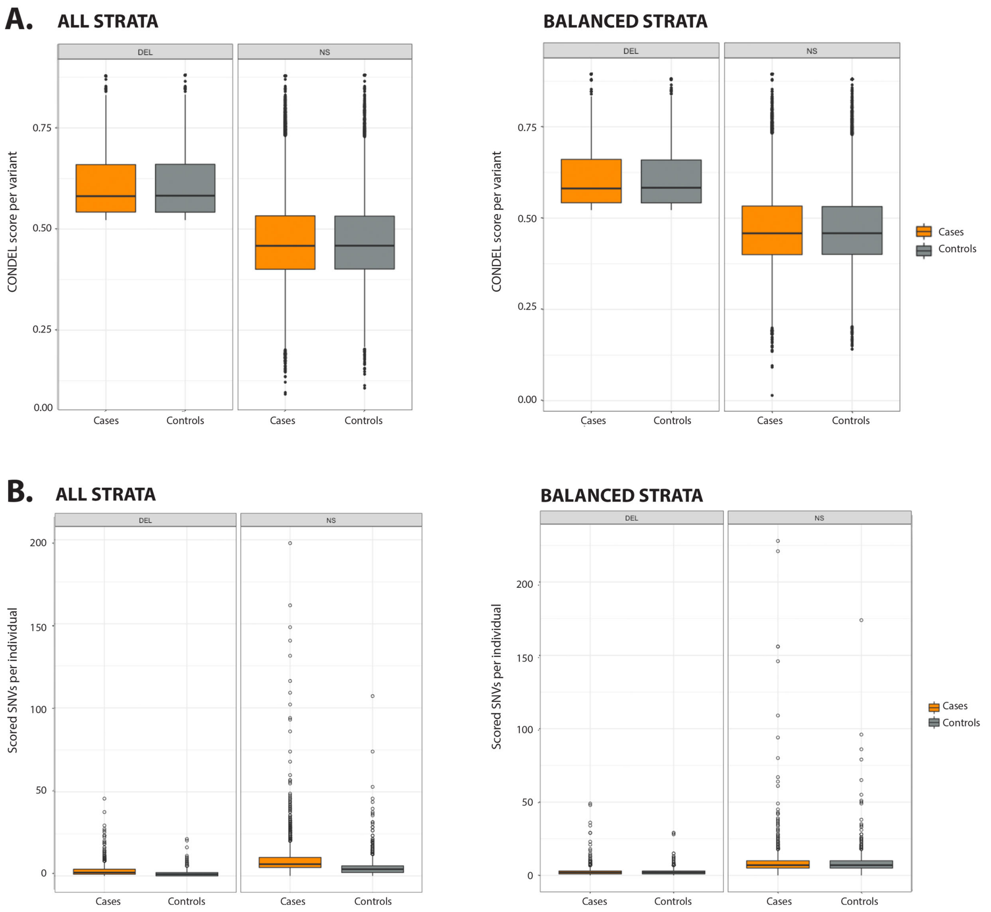

Supplementary Figure 6. Power plot of single variant analysis.

Results derived from Genetic Power Calculator (Purcell *et al*., 2003). Grey line corresponds to minimal minor allele frequency (MAF) and genotype relative risk needed at these sample sizes to obtain at least 80% power for single-locus tests at a significance level of $5x10^{-7}$. Dotted orange lines depict MAF cut-offs at 5%, 1% and 0,1%.

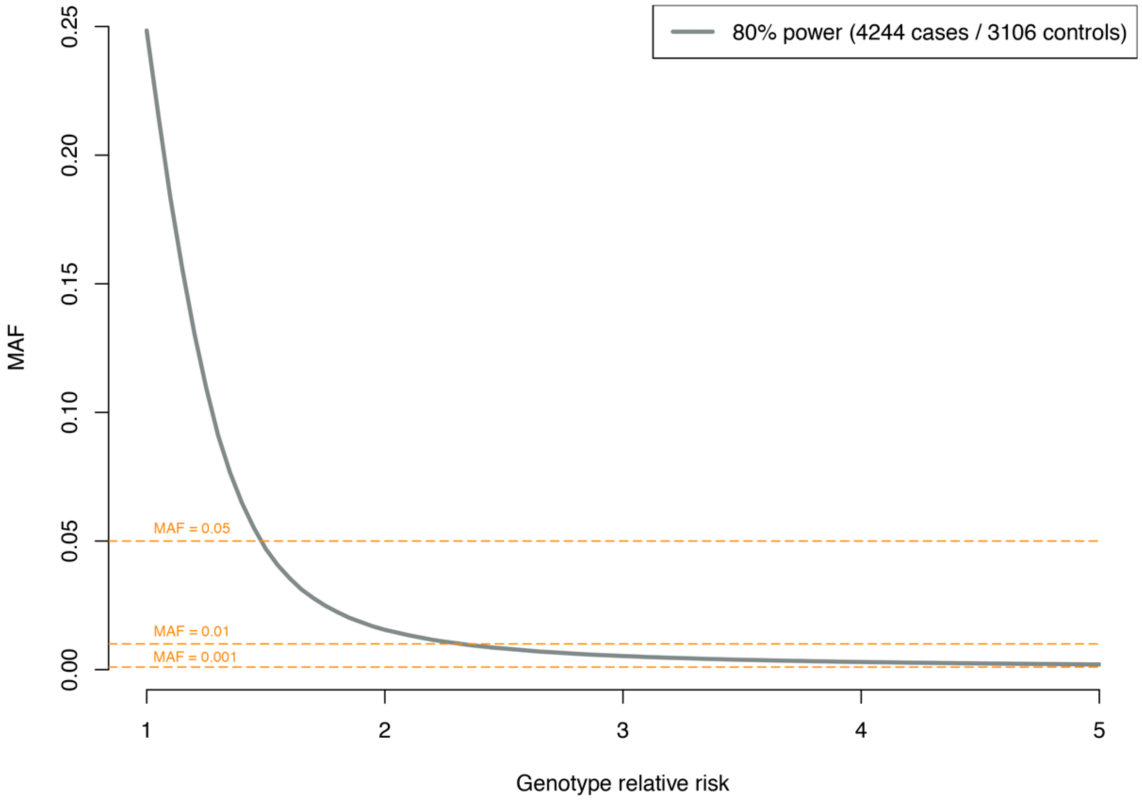

Supplementary Figure 7. Power plot of gene-based burden test

Since the specific genetic architecture of ALS is still unknown, power analysis for gene-based burden test (SKAT-O) as implemented in the R-package SKAT under different genic architectures (different percentages of causal (10%, 20%, 30%, 40% and 50%) and protective (0%, 10%, 20%, 30%, 40% and 50%) variants contributing to the signal) at significance level of $3.44x10^{-6}$. Number of simulations: 200). The term 'causal percent' refers to the percentage of rare variants (which we defined as MAF < 0.01) in a genomic region assumed to be causal, i.e. risk-increasing. The term 'protective percent' refers to the percent of rare variants assumed to have a negative coefficient. The assumed maximum OR is set at 5 when MAF=0.0001 (default setting).

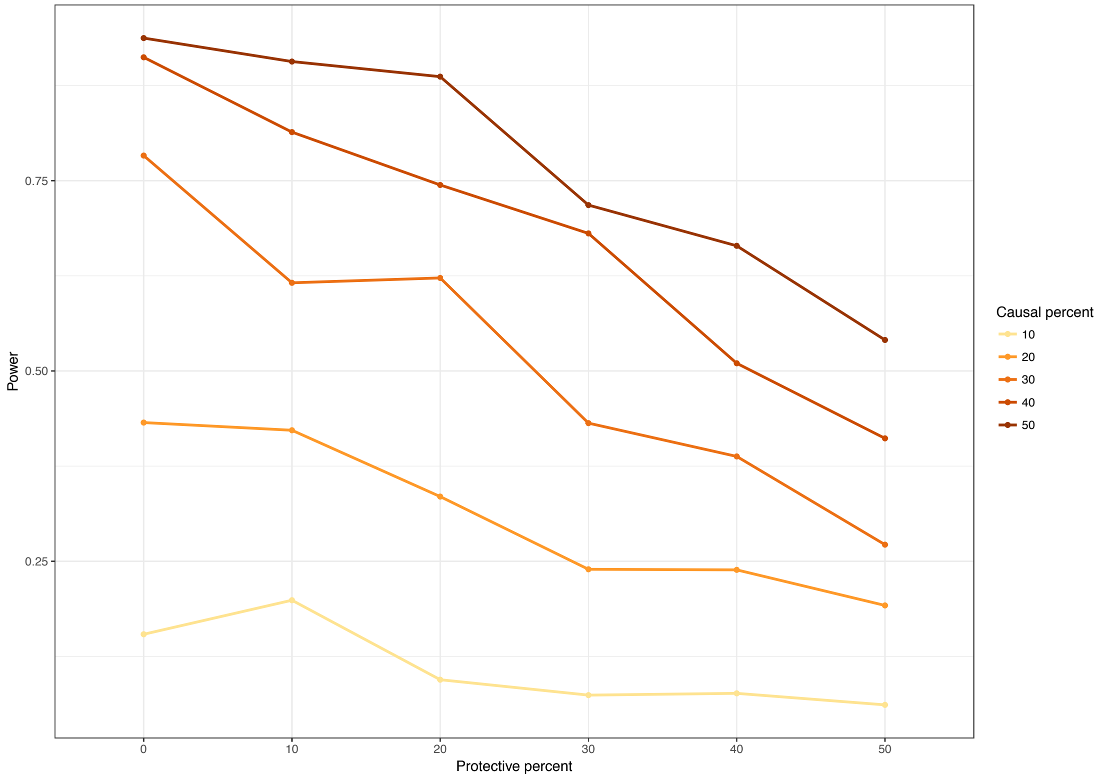

Supplementary Figure 8. PCA plots of population structure.

A. Data projected onto principal components calculated on individuals from HapMap 3. Population outliers are defined as ±10 standard deviations from the HapMap CEU mean on PC1–PC4.

B. Data projected onto principal components calculated on individuals from The 1000 Genome Project after removal of HapMap 3 population outliers. Population outliers are defined as ±10 standard deviations from the 1000 Genome CEU mean on PC1–PC4.

A.

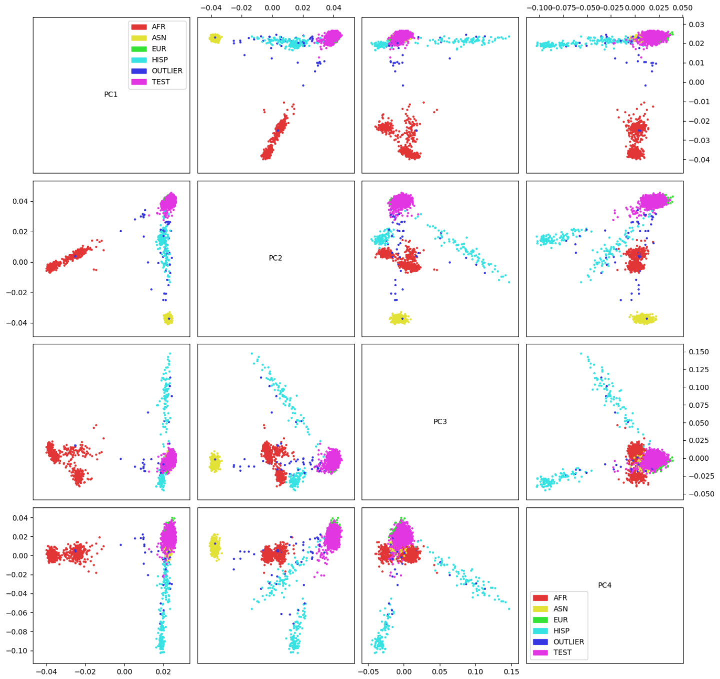

B.

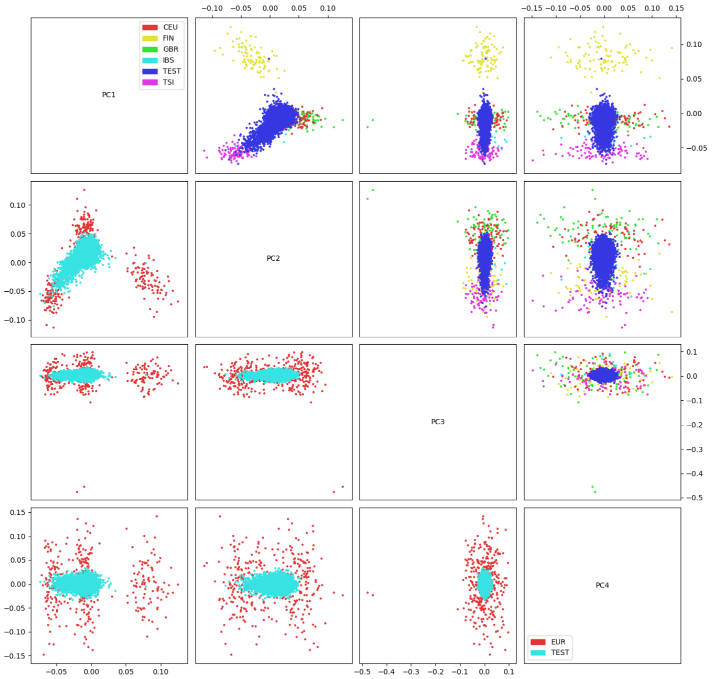

Supplementary Table 1. Study population.

Overview of included samples from seven different sites across six European countries after quality control.

Country	Site	Cases	Controls	Total
The Netherlands	Utrecht	1611	1693	3304
Belgium	Leuven	472	485	957
Germany	Wurzburg / Ulm	1269	343	1612
Ireland	Dublin	406	402	808
Spain	Madrid	188	87	275
Italy	Turin	298	96	394
Total		***4244***	***3106***	***7350***

Supplementary Table 2. Single nucleotide variant association test results using logistic regression.

Results limited to top-10 associations. Positions given for human build 37. ExAC minor allele frequencies derived from ExAC browser, European non-Finnish population. Annotation based on Ensembl Variant Effect Predictor. OR = odds ratio, CI = confidence interval, MAF = minor allele frequency.

RS number	Chr	Position (bp)	OR (95% CI)	p value	MAF case (%)	MAF con (%)	MAF ExAC (%)	Annotation	Gene
rs3849942	9	27543281	1.21 (1.12 – 1.31)	1.72 $x10^{-6}$	0.267	0.230	/	Downstream gene	*C9orf72*
rs2814707	9	27536397	1.19 (1.10 – 1.29)	9.74 $x10^{-6}$	0.267	0.233	/	Intergenic	
rs73168055	7	149481994	1.38 (1.19 – 1.60)	2.25 $x10^{-5}$	0.068	0.050	0.059	Non-coding transcript	*SSPO*
rs200161705	4	170506525	2.74 (1.68 – 4.47)	5.76 $x10^{-5}$	0.009	0.003	0.004	Missense	*NEK1*
rs1871332	5	101019725	0.82 (0.74 – 0.90)	6.52 $x10^{-5}$	0.129	0.154	/	Intergenic	
rs774359	9	27561049	1.17 (1.08 – 1.26)	8.77 $x10^{-5}$	0.286	0.255	/	Intron	*C9orf72*
rs3743797	16	52478215	1.28 (1.13 – 1.46)	1.00 $x10^{-4}$	0.090	0.073	0.136	Synonymous	*TOX3*
rs12929114	16	65532244	1.14 (1.07 – 1.23)	1.50 $x10^{-4}$	0.429	0.393	/	Intron	*LINC00922*
rs181906086	2	31414830	0.35 (0.20 – 0.60)	1.68 $x10^{-4}$	0.003	0.006	0.004	Missense	*CAPN14*
rs2715148	7	82450035	0.88 (0.82– 0.94)	1.72 $x10^{-4}$	0.472	0.512	/	Intron	*PCLO*

Supplementary Table 3. Single nucleotide variant association test results for previously identified ALS variants

Single variant association results for variants previously associated with ALS (Van Rheenen *et al*, 2016). Positions given for human build 37. Nearest gene or previously published gene names are provided. 'Number of variants in gene' refers to the number of variants annotated to the genic region, based on the Illumina gene annotation file. 'Number of variants tested' refers to the number of polymorphic variants in the genic region in our study.

Variant information				*Van Rheenen et al. 2016 (12,577 cases / 23,475 controls)*		*Current study*				
**SNP**	**Chr**	**Position (bp)**	**Gene**	**OR (95% CI)**	**p value**	**Present on exome array**	**OR (95% CI)**	**p value**	**# Variants in gene**	**# Variants tested**
rs616147	3	39534481	*MOBP*	1.10 (1.06 – 1.13)	4.2×10^{-10}	No	/	/	6	5
rs3849942	9	27543281	*C9orf72*	1.19 (1.15 – 1.23)	3.8×10^{-10}	Yes	1.21 (1.12 – 1.31)	1.72×10^{-6}	5	2
rs74654358	12	31147498	*TBK1*	1.21 (1.13 – 1.3)	6.6×10^{-8}	No	/	/	11	5
rs10139154	14	31147498	*SCFD1*	1.09 (1.05 – 1.13)	3.5×10^{-8}	No	/	/	4	2
rs35714695	17	26719788	*SARM1*	0.86 (0.84 – 0.91)	9.0×10^{-11}	No	/	/	11	1
rs12608932	19	17752689	*UNC13A*	1.10 (1.07 – 1.14)	2.7×10^{-10}	No	/	/	17	8
rs75087725	21	45753117	*C21orf2*	1.45 (1.28 – 1.65)	3.1×10^{-10}	No	/	/	12	6

Supplementary Table 4. Variant characteristics gene-based burden test *NEK1* and *CAPN14*.

Variant characteristics and results for SKAT-O gene-based burden test for *NEK1* and *CAPN14* after conditioning on the top hit from the single variant analysis (flagged with *). Positions given for human build 37. MAF; minor allele frequency

Exome array ID	Chr	Position (bp)	Gene	MAF case (%)	MAF con (%)	SKAT-O p value	Conditioned p value*
exm184273	2	31399482	*CAPN14*	0.0002356	0.000322		
exm184287	2	31401419	*CAPN14*	0.0007069	0.0006441		
exm184311*	2	31414830	*CAPN14*	0.002593	0.00628	$1.73\ x10^{-4}$	0.70
exm184315	2	31414919	*CAPN14*	0.0007069	0.001127		
exm184329	2	31420161	*CAPN14*	0.001649	0.001932		
exm433918	4	170398474	*NEK1*	0.007069	0.004834		
exm433919	4	170398485	*NEK1*	0.0004715	0.0006441		
exm433923	4	170398627	*NEK1*	0.0001178	0.000161	$1.21\ x10^{-5}$	0.12
exm433965*	4	170506525	*NEK1*	0.008836	0.003381		
exm433966	4	170506526	*NEK1*	0.0002356	0		
exm433970	4	170506621	*NEK1*	0.0002356	0		

Supplementary Table 5. Exome array resolution for previously identified ALS genes

Resolution of the exome array for previously associated ALS susceptibility genes compared to variant data from the ExAC Browser (missense / LOF variants in ExAC canonical transcripts, obtained from http://exac.broadinstitute.org/). 'Number of variants on array' refers to the number of variants annotated to the gene based on based on the Illumina gene annotation file. 'Number of variants in genic burden test' refers to the number of variants included in SKAT-O gene-based analysis after QC and annotation based on Ensembl Variant Effect Predictor. 'Number of variants in ALS domains' refers to the variants in ALS-associated domains for the genes *FUS*, *TARDBP* and *KIF5A*, since ALS associated variation in these genes is highly region specific. These regions refer to *TARDBP* exon 6, *FUS* exons 5, 6, 14 and 15 and *KIF5A* exons 24 – 28.

		ExAC		*Current study*			
**Gene**	**Chr**	**MAF > 0.01**	**MAF < 0.01**	**# variants on array**	**# variants in genic burden test**	**# variants in ALS domains**	**SKAT-O p value**
SOD1	21	0	25	2	0	/	/
FUS	16	0	162	5	1	1	0.72
TARDBP	1	0	37	1	1	0	0.89
KIF5A	12	1	200	7	3	1	0.59
NEK1	4	3	402	23	6	/	2.73×10^{-5}
C21orf2	21	4	155	12	3	/	0.82

Supplementary Table 6. Comparison of set-unique variant count per individual.

Results given for analysis comprising all individuals (all cohorts; N = 7350) and for a subset of samples comprising balanced case-control cohorts only (balanced cohorts; samples from The Netherlands, Belgium and Ireland, N = 5069). P values given for Wilcoxon rank sum test. DEL = deleterious variants, NS = all non-synonymous and loss-of-function variants, sd = standard deviation, ind = individuals.

		**ALL COHORTS**				**BALANCED COHORTS**			
**Variant type**	**Phenotype**	**No. of individuals**	**No. of SNVs**	**Scored SNVs / ind (mean / median / sd)**	**p value**	**No. of individuals**	**No. of SNVs**	**Scored SNVs / ind (mean / median / sd)**	**p value**
**DEL**	*Case*	4244	6600	2.56 / 2 / 2.55	2.89×10^{-150}	2489	4201	2.36 / 2 / 2.62	0.33
	Control	3106	3135	1.36 / 1 / 1.50		2580	4025	2.28 / 2 / 1.91	
**NS**	*Case*	4244	23,231	9.08 / 7 / 8.96	1.38×10^{-276}	2489	14,831	8.33 / 7 / 9.88	0.19
	Control	3106	11,088	4.80 / 4 / 4.48		2580	14,310	7.99 / 7 / 6.09	

Supplementary Table 7. Comparison of CONDEL score per scored variant

Results given for analysis comprising all individuals (N = 7350) and for a subset of samples comprising balanced case-control cohorts only (samples from The Netherlands, Belgium and Ireland, N = 5069). P values given for Wilcoxon rank sum test. DEL = deleterious variants, NS = all non-synonymous and loss-of-function variants, sd = standard deviation, SNV = single nucleotide variant.

		**ALL COHORTS**				**BALANCED COHORTS**			
**Variant type**	**Phenotype**	**No. of individuals**	**No. of SNVs**	**CONDEL score / SNV (mean / median / sd)**	**p value**	**No. of individuals**	**No. of SNVs**	**CONDEL score / SNV (mean / median / sd)**	**p value**
**DEL**	*Case*	4244	6,600	0.62 / 0.58 / 0.10	0.28	2489	4201	0.62 / 0.58 / 0.11	0.86
	Control	3106	3,135	0.62 / 0.58 / 0.11		2580	4025	0.62 / 0.58 / 0.10	
**NS**	*Case*	4244	23,231	0.48 / 0.46 / 0.12	0.58	2489	14,831	0.48 / 0.46 / 0.12	0.87
	Control	3106	11,088	0.48 / 0.46 / 0.12		2580	14,310	0.48 / 0.46 / 0.12	
